# Molecular docking study and antiviral evaluation of 2-thioxo-benzo[*g*]quinazolin-4(3*H*)-one derivatives

**DOI:** 10.1186/s13065-016-0168-x

**Published:** 2016-04-19

**Authors:** Rashad Al-Salahi, Hatem A. Abuelizz, Hazem A. Ghabbour, Rabab El-Dib, Mohamed Marzouk

**Affiliations:** Department of Pharmaceutical Chemistry, College of Pharmacy, King Saud University, P. O. Box 2457, Riyadh, 11451 Saudi Arabia; Department of Pharmacognosy, College of Pharmacy, King Saud University, P.O. Box 22452, Riyadh, 11495 Saudi Arabia; Department of Pharmacognosy, Faculty of Pharmacy, Helwan University, Cairo, 11795 Egypt; Chemistry of Natural Products Group, Center of Excellence for Advanced Sciences, National Research Center, Dokki, Cairo, 12622 Egypt

**Keywords:** 2-Thioxo-benzo[*g*]quinazolines, HSV, Coxsackievirus, Molecular docking, Ribavirin

## Abstract

**Background:**

The persistent appearance of viral strains that causes a resistant viral infection has led to continuous trials for the design and development of novel antiviral compounds. Benzoquinazoline compounds have been reported to exhibit an interesting antiviral activity. This work aims to study and evaluate the antiviral activity of a newly prepared 2-thioxo-benzo[*g*]quinazolin-4(3*H*)-one series against herpes simplex (HSV-1 & 2) and coxsackievirus (CVB4).

**Methods:**

The antiviral activity was performed using the MTT assay, in which Vero cells (obtained from the American Type Culture Collection, ATCC) were propagated in fresh Dulbecco’s Modified Eagle’s Medium (DMEM) and challenged with 10^4^ doses of the virus. Thereafter, the cultures were treated simultaneously with two-fold serial dilutions of the tested compound and incubated at 37 °C for 48 h. Molecular docking studies were done on the CVB4 2A proteinase enzyme using Molegro Virtual Docker software.

**Results:**

The cytotoxicity (CC_50_), effective concentration (EC_50_) and the selectivity index (SI) values were determined. Based on their EC_50_ values, a number of the investigated compounds demonstrated weak to moderate activity relative to their parents. Accordingly, compounds **5**–**9**, **11**, **15**–**18**, **21**, **22**, **24**, **25**, **27** and **28** were active against CVB4, and compounds **5** and **24** were active against HSV-1 and 2 in comparison to ribavirin and acyclovir, which were used as reference drugs.

**Conclusion:**

The obtained results gave us some useful insights about the characteristic requirements for future trials to build up and design more active and selective antiviral 2-thioxo-benzo[*g*]quinazolin-4(3*H*)-one agents.

**Graphical abstract:**
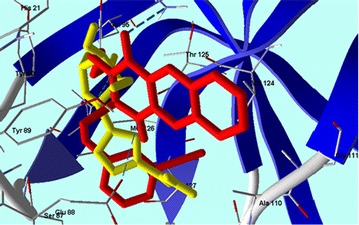
Compound **24** superimposed with Ribavirin in CV B4 2A Proteinase enzyme (PDB: 1Z8R) active site.

## Background

Herpes simplex (HSV-1 & 2) and Coxsackie B4 (CVB4) viruses belong to the *alphaherpesvirinae* and *picornaviridae* families, respectively. In contrast to HSV-1 and 2 which classified as enveloped double-stranded DNA viruses, CVB-4 is non-enveloped RNA viruses. They are common human pathogens and considered a significant worldwide health concern [1–3]. A relatively wide range of diseases, ranging from asymptomatic, mild infections to serious illnesses, are caused by these viruses [4, 5]. In addition, infections by CVB4 have also been known to cause aseptic meningitis, encephalitis, pleurodynia, myocarditis, and pericarditis [5].

Viral infectious diseases pose a major challenge for modern medicaments because the viruses have high mutation rates, which allow them to escape immune systems and become resistant to the traditional antiviral drugs [6–10]. Furthermore, although the antiviral drugs for diseases caused by several types of viruses such as herpes are available clinically, but the high prevalence of viral infections for which there are no specific treatments or the continuous appearance of new resistant viral strains are serious problems. This make the task of the development of new novel antiviral agents is essential [10].

Recently, we have reported the biological activity of some prepared triazoloquinazolines against herpes simplex (HSV-1 & 2) and CVB4. However, a number of these prepared compounds were found to possess remarkable and significant antiviral activity [11–13]. Furthermore, synthetic chemistry has shown that benzoquinazoline is a valuable precursor for elaborating many structurally diverse bioactive molecules, particularly as influenza H5N1 and H1N1 antiviral agents [14–17]. In addition, some 2-aminobenzo[*de*]-isoquinoline-1,3-diones have been reported as antiherpetic agents [11].

In view of these evidences and an extension of our ongoing research on benzoquinazolines chemistry, we herein report the antiviral evaluation of a new series of 2**-**thioxo-benzo[*g*]quinazolin-4(3*H*)-one derivatives against HSV-1, HSV-2 and CVB4 viruses.

## Results and discussion

**Scheme 1:**
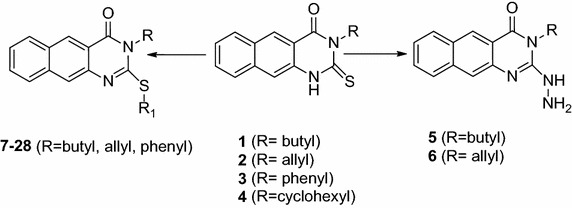
Synthetic route for 2-thioxo-benzo[*g*]quinazolines (**1**–**28**)

We previously reported our findings regarding the antiviral activity of isoquinazoline and triazoloquinazoline derivatives. The results suggested that quinazolines can be good platform for designing a new antiviral agent [11–13]. Here, we are reporting the results of an antiviral investigation for a new series of 2-thioxo-benzo[*g*]quinazolines **1**–**28** (Table 1 and Scheme 1) [18]. The evaluation of the synthesized compounds **1**–**28** against HSV-1, HSV-2 and CVB4 was assessed in vitro using an MTT assay. Their cytotoxic effects were also evaluated. Results obtained from this screening showed that most of the compounds demonstrated antiviral activity, which ranged from weak through moderate to high effects, based on EC_50_ and SI values relative to their parent and reference drugs (Table 2). In accordance to the statistical analyses and in terms of SI as a marker for antiviral activity, all tested molecules have been classified into three groups: inactive- (SI < 2), active- (2 ≤ SI < 10) and very active-types (SI ≥ 10) [19]. Accordingly, compounds **5**–**9**, **11**, **15**–**18**, **21**, **22**, **24**, **25**, **27** and **28** were active against CVB4. On the other hand, compound **5** has shown activity against HSV 1 and 2, while **24** was found to be active against HSV 1. It may be noticed that the tested molecules **5** and **9** showed significant levels of high activity against CVB4, with SI values of 6.27 and 5.77, whereas **15**, **21** and **24** were less active (3.60, 3.73 and 3.85, respectively) with regard to ribavirin (16.38). However, **6**, **7**, **8**, **11**, **16**, **17**, **18**, **22**, **25**, **27** and **28** exhibited moderate activity against CVB4, with SI values in the range of 2.05‒3.31. Moreover, compound **5** demonstrated good activity against HSV-1 and HSV-2 (SI = 4.28 and 5.18, respectively) and **24** was active against HSV-1 (SI = 2.61) in relation to ribavirin (41.93 and 24.69).

**Table 1:** Synthesized 2-thioxo-benzo[*g*]quinazolines (**7**–**28**)

CPs	R	R_1_	CPs	R	R_1_
**7**	Butyl	Ethyl	**18**	Allyl	3-methoxybenzyl
**8**	Butyl	Allyl	**19**	Allyl	4-chlorobenzyl
**9**	Butyl	Benzyl	**20**	Allyl	2-morpholinoethyl
**10**	Butyl	3-methoxybenzyl	**21**	Allyl	3-(phthalimido-2-yl)propyl
**11**	Butyl	4-chlorobenzyl	**22**	Phenyl	Ethyl
**12**	Butyl	4-cyanobenzyl	**23**	Phenyl	Allyl
**13**	Butyl	2-piperidinoethyl	**24**	Phenyl	3-cyanobenzyl
**14**	Butyl	2-morpholinoethyl	**25**	Phenyl	4-chlorobenzyl
**15**	Butyl	3-(phthalimido-2-yl)propyl	**26**	Phenyl	2-piperidinoethyl
**16**	Allyl	Ethyl	**27**	Phenyl	2-morpholinoethyl
**17**	Allyl	Allyl	**28**	Phenyl	3-(phthalimido-2-yl)propyl

**Table 2:** Antiviral activity against HSVand CVB4 of compounds (**1**–**28**) in terms of CC_50_, EC_50_ (μg/mL) and SI

Cpd Nr.	CC_50_	HSV-1		HSV-2		CVB4	
		EC_50_	SI	EC_50_	SI	EC_50_	SI
**1**	115.9	Inactive	Inactive	Inactive	Inactive	272.4	0.43
**2**	173.1	247.5	0.70	213.8	0.81	149.2	1.16
**3**	376.5	428.1	0.88	361.6	1.04	342.4	1.10
**4**	824.7	706.4	1.17	649.2	1.27	478.9	1.72
**5**	3840	896.4	4.28	740.8	5.18	612.8	6.27
**6**	261.4	224.5	1.16	208.9	1.25	102.9	2.54
**7**	105.7	73.8	1.43	81.4	1.30	38.9	2.72
**8**	218.5	147.1	1.49	159.6	1.37	93.6	2.33
**9**	546.9	316.5	1.73	359.2	1.52	94.8	5.77
**10**	198.9	162.7	1.22	184.3	1.08	124.1	1.60
**11**	542.6	403.9	1.34	467.3	1.16	216.2	2.51
**12**	83.7	74.12	1.13	65.9	1.27	56.4	1.48
**13**	652.4	594.6	1.10	681.3	0.96	371.8	1.75
**14**	221.8	197.3	1.12	176.4	1.26	116.8	1.90
**15**	376.2	243.2	1.55	260.8	1.44	104.6	3.60
**16**	934.2	582.9	1.60	624.6	1.50	316.4	2.95
**17**	132.7	81.2	1.63	104.8	1.27	50.2	2.64
**18**	183.4	149.8	1.22	140.5	1.31	89.5	2.05
**19**	236.5	189.3	1.25	174.9	1.35	157.1	1.51
**20**	465.3	369.1	1.26	402.9	1.15	287.6	1.62
**21**	968.7	674.8	1.44	812.6	1.19	259.4	3.73
**22**	127.3	76.3	1.67	89.4	1.42	41.2	3.09
**23**	205.6	183.6	1.12	165.2	1.24	149.2	1.38
**24**	169.1	64.7	2.61	112.3	1.51	43.9	3.85
**25**	431.6	307.2	1.40	284.8	1.52	204.3	2.11
**26**	681.2	498.2	1.37	514.6	1.32	395.6	1.72
**27**	1034.8	>1000	Inactive	>1000	Inactive	475.3	2.17
**28**	189.6	162.4	1.17	178.6	1.06	57.3	3.31
**Acyclovir**	648.2	2.3	281.83	1.06	144.04	–	–
**Ribavirin**	486.4	11.6	41.93	11.3	24.69	29.7	16.38

Cells treated with DMSO (0.1 %) were used as a negative control, and its reading was subtracted from the readings of tested compounds. Statistics were calculated using one-way ANOVA

**Fig. 1:**
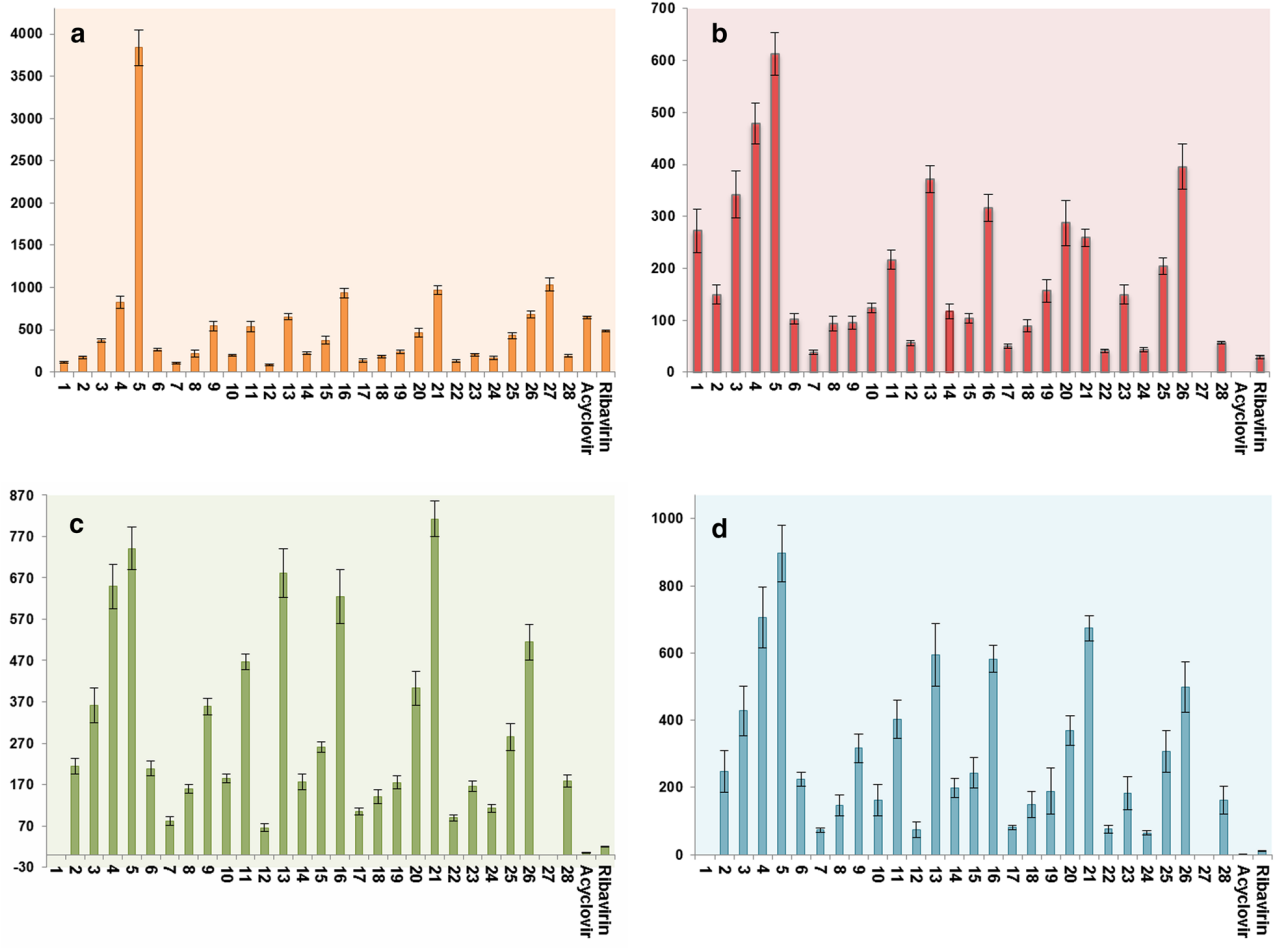
Antiviral and cytotoxicity evaluation of the synthesized compounds **1**–**28** compared to ribavirin and acyclovir. **a** Cytotoxicity effect (CC_50_). **b** Antiviral evaluation against CVB4 (EC_50_). **c** Antiviral evaluation against HSV-2 (EC_50_). **d** Antiviral evaluation against HSV-1 (EC_50_). All the values represented in (μg/mL)

In outlining the results in Table 2 and Fig. 1, it should be clarified that modifications on the lead structures **1**–**3** afforded new structural features (**5**–**28**) with a wide range of effects against the HSV and CVB4 viruses. For instance, *S*-alkylated products **7**–**28** exhibited significant activity against Coxsackie B4. In particular, compounds **7**–**9**, **11**, **15**–**18**, **21**, **22**, **24**, **25**, **27** and **28** were more active than their parents **1**–**3**. Moreover, variations in the type of the *N*-alkyl and *S*-alkyl (heteroalkyl) groups resulted in variations of the activity, in which compound **9** represented against CVB4 as the most active among the *S*-alkylated compounds (SI = 5.57). Compounds **15**, **21**, **24** and **28** showed a pronounced activity against CVB4 (SI = 3.60, 3.73, 3.85 and 3.31, respectively). In regard to anti-herpes activity, compound **1** was inactive, but its *S*-alkylated products **7**–**15** exhibited slight activity. Similarly, the parents **2** and **3** appeared less active than their chemically transformed products **16**–**21** and **22**–**28**, respectively. However, hydrazino products **5** and **6** offered more advantages in terms of activity against HSV and CVB4 viruses. Depending on the values of the SI-parameter, **5** gave rise to the greatest activity against HSV-1 (4.28), followed by HSV-2 (5.18) and CVB4 (6.27). Moreover, the presence of the butyl group at the “R” position provided a significant effect against CVB4 and HSV viruses. This effect can be seen in both *S*-alkylated and hydrazino derivatives. However, the “R_1_” position requires a hydrophobic moiety to provide a selective antiviral activity against CVB4, as in compound **9**. On the other hand, compound **5** exhibited a non-specific antiviral activity against CVB4 and HSV viruses. This effect also can be seen with compound **24** that has a 3-cyanobenzyl moiety at “R_1_” position but with a phenyl group instead of butyl at “R” position.

**Fig. 2:**
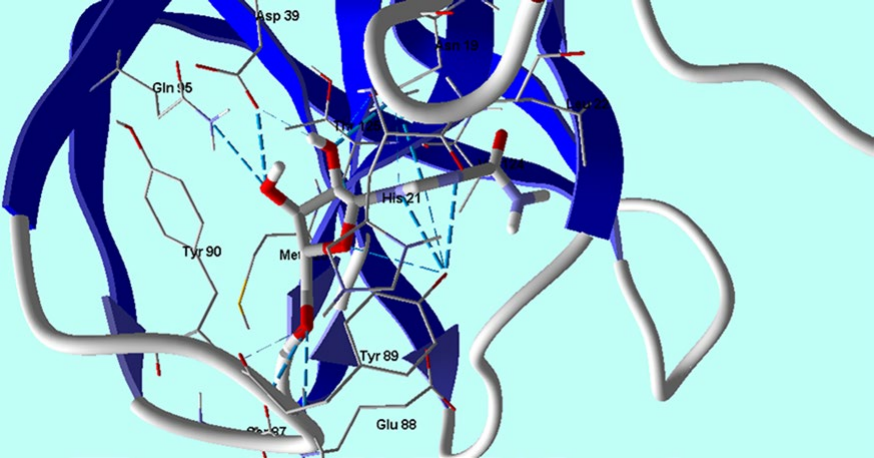
Ribavirin shows hydrogen bonds interactions with CVB4 2A Proteinase enzyme (PDB: 1Z8R) active site

To investigate the effect of the different variation of the original skeletons, a molecular docking experiment has been done with correlation to CVB4 2A proteinases. CVB4 2A proteinases perform essential roles involving viral polyprotein self-processing and shutting down of host-cell protein synthesis during viral replication. In addition, CVB4 2A proteinases also cleave heart muscle dystrophin, leading to cytoskeletal dysfunction and the symptoms of human-acquired dilated cardiomyopathy [20]. In silico docking experiments were performed for compounds **1**–**28** against the X-ray crystal structure of Coxsackievirus B4 2A proteinases (Protein Data Bank (PDB): 1Z8R) [20] using Molegro Virtual Docker software. Docking results were then evaluated by the MolDock score function, and hydrogen bond interactions between tested compounds and the target receptor were used for comparison between the tested and reference compounds [21]. Ribavirin (reference drug) forms eleven hydrogen bonds with amino acid residues at the active site: Tyr 89, Asn 19, Glu 88, Gln 95, Asp 39 and Thr 125, and generated a MolDock score of –100.84 (Fig. 2).

**Fig. 3:**
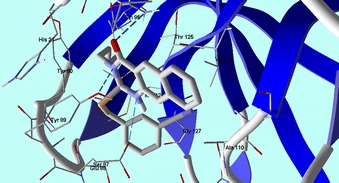
Compound **24** shows hydrogen bonds interactions with CVB4 2A Proteinase enzyme (PDB: 1Z8R) active site

**Fig. 4:**
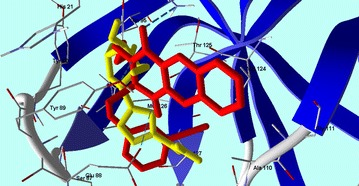
Compound **24** superimposed with Ribavirin in CV B4 2A Proteinase enzyme (PDB: 1Z8R) active site

Compounds **1**–**6** had MolDock scores ranging from −81.42 to −84.81 (Table 3). These scores increased from −84.82 to −126.89 in compounds **7**–**28**, and reached the highest levels (−124.852, −124.156 and −126.899) in compounds **10**, **18** and **24**, respectively. However, compounds **10** and **18** have a 3-methoxybenzyl group at the “R_1_” position, but they are varied between each other with butyl group in compound **10** and allyl group in compound **18** at the “R” position. Even though, their MolDock scores were high but it did not enhance their antiviral activity. On the other hand, compound **24** that gave the highest MolDock score in this experiment has a phenyl group at “R” position and 3-cyanobenzyl group at “R_1_” position. Compound **24** made three hydrogen bonds with the amino acid residues (Tyr 89, Asn 19 and Glu 88) with CVB4 2A Proteinase enzyme (PDB: 1Z8R) active site (Fig. 3). Interestingly, the *para* position of “R_1_” substituted benzyl group, such as compound **12**, did not enhance the MolDock score than the *meta* position as in compound **10** and **18**. This supports the notion that a hydrophobic moiety at the “R” position is important for the protein binding and the wide range of antiviral activity against CVB4 and HSV. We propose that the phenyl group in compound **24** might participate in a non-polar staking interaction. Moreover, the quality of the docking process was attributed to the good overlapping of compound **24** with ribavirin in the active site (Fig. 4). Taking into account the preceding results, *S*-alkylated products **7**–**28** demonstrated good interaction with CVB4 with regard to the parent compounds (**1**–**3**), along with **9**, **21** and **24** that indicate good relation with the biological results in Table 3.

**Table 3:** Molecular docking results of tested compounds (**1**–**28**)

Ligand	MolDock score	Rerank score	Ligand	MolDock score	Rerank score
**1**	−84.7301	11.9741	**15**	−102.661	170.385
**2**	−81.6688	−54.7013	**16**	−89.6801	−49.3148
**3**	−83.1126	−64.3716	**17**	−99.0106	−61.5796
**4**	−81.4295	−47.6937	**18**	−124.156	−35.6187
**5**	−84.4966	−61.7217	**20**	−108.311	3.82767
**6**	−84.8156	−57.6283	**21**	−97.9703	146.694
**7**	−97.1415	−28.781	**22**	−93.5541	−43.7713
**8**	−106.264	−13.9656	**23**	−86.1706	16.8665
**9**	−109.555	16.3502	**24**	−126.899	−16.0488
**10**	−124.852	−41.5862	**25**	−101.643	−45.7134
**11**	−101.561	−24.4802	**26**	−102.852	15.5337
**12**	−112.213	10.1418	**27**	−106.807	−8.86485
**13**	−98.2456	2.8343	**28**	−84.8292	52.4014
**14**	−98.3327	−16.7182	**Ribavirin**	−100.849	−68.7835

## Methods

### Mammalian cell line

The source and methodology for preparation of the Vero cells were reported in details by Al-Salahi et collaborators [11]. The GHSV-UL46, G and E2 viral strains were used for the assay of HSV-1, HSV-2 and CVB4 viruses, respectively.

### Evaluation of the antiviral activity

Screening of the antiviral was performed using MTT assay. According to the literature [11, 22, 23], the Vero cells were cultured, then treated with two-fold serial dilutions of the tested compounds, starting from 1000 μg/mL and diluting to about 2 μg/mL (1000, 500, 250, 125, 62.5, 31.25, 15.63, 7.81, 3.91, 1.95 μg/mL). Six wells were used for each concentration of the tested compound and three independent experiments were assessed, each containing four replicates per treatment [24]. Untreated Vero cell control and infection controls were made in the absence of tested compounds. Acyclovir and ribavirin were used as positive controls in this assay [25].

After incubating for 48 h, the numbers of viable cells were determined by the MTT test. Briefly, the medium was removed from the 96-well plate and replaced with 100 μL of fresh RPMI 1640 medium without phenol red, then 10 μL of the 12 mM MTT stock solution [5 mg of MTT in 1 mL of phosphate-buffered saline (PBS)] to each well, including the untreated controls. The 96-well plates were then incubated at 37 °C and 5 % CO_2_ for 4 h. An 85 μL aliquot of the medium was removed from the wells, and 50 μL of dimethyl sulfoxide (DMSO) were added to each well, mixed thoroughly with the pipette, and incubated at 37 °C for 10 min. Then, the optical density was measured at 590 nm with a microplate reader (Sunrise, Tecan U.S. Inc., USA) to determine the number of viable cells [11, 22, 26].

The viral inhibition rate was calculated as follows:

$${\text{Viral Inhibition Rate }} = \left[ {{{\left( {{\text{ODtv }} - {\text{ ODcv}}} \right)} / {\left( {{\text{ODcd}} - {\text{ ODcv}}} \right)}}} \right] \, \times \, 100\;\% ,$$

where ODtv, ODcv and ODcd indicate the absorbance of the tested compounds with virus-infected cells, the absorbance of the virus control and the absorbance of the cell control, respectively. The EC_50_ was estimated with respect to the virus control from the graphic plots, using STATA modelling software and (SI) calculated from the ratio of CC_50_ to EC_50_ [11, 26].

### Cytotoxicity evaluation using viability assay

The procedure for seeding and incubation of Vero cells was explained in details in previous research [11, 23, 27]. After the end of the incubation period, the number of viable cells was determined by the MTT test. Briefly, the medium was removed from the 96-well plate and replaced with 100 μL of fresh RPMI 1640 medium without phenol red, then 10 µL of the 12 mM MTT stock solution (5 mg of MTT in 1 mL of PBS) to each well including the untreated controls. The 96-well plates were then incubated at 37 °C and 5 % CO_2_ for 4 h. An 85 μL aliquot of the medium was removed from the wells, and 50 μL of DMSO were added to each well, mixed thoroughly with the pipette, and incubated at 37 °C for 10 min. Then, the optical density was measured at 590 nm with the microplate reader (Sunrise, Tecan U.S. Inc., USA) to determine the number of viable cells. Without added stain, all obtained findings were corrected for background absorbance detected in wells. In the absence of the tested compounds, treated samples were compared with the cell controls. All experiments were carried out in triplicate. The cytotoxicity of each tested compound was calculated [24, 25, 27, 28].

The percentage cell viability, calculated using Microsoft Excel^®^, is as follows:

$$\% {\text{ Cell Viability }} = \left[ {{{\left( {{\text{Mean Abs}}_{\text{control}} - {\text{Mean Abs}}_{\text{test metabolite}} } \right)} \mathord{\left/ {\vphantom {{\left( {{\text{Mean Abs}}_{\text{control}} - {\text{Mean Abs}}_{\text{test metabolite}} } \right)} {{\text{Mean Abs}}_{\text{control}} }}} \right. \kern-0pt} {{\text{Mean Abs}}_{\text{control}} }}} \right] \, \times { 1}00\;\% ,$$

where Abs equals the absorbance at 590 nm. The STATA statistical analysis package was used for the dose response curve, which was used to calculate CC_50_.

### Data analysis

Statistical analysis was done using a one-way ANOVA test [29]. All experiments and data analysis of the antiviral and cytotoxicity evaluations were carried out in RCMB, Al-Azhar University, Cairo, Egypt.

### Molecular docking

The modelling studies were done by a PC with Intel© Core™ i7-3630 QM CPU (2.40 GHz, RAM 8 GB) operating under the Windows 7 Professional Operating System [11]. The modelling processes included several steps: first, download the 3D crystal structures of the Coxsackievirus B4 2A proteinase enzyme with PDB code 1Z8R (Brookhaven Protein Data) [20], and then load this into the Molegro Virtual Docker (MVD 2013.6.0 [Win32]) program (fully functional, free trial version with time limiting license; Molegro Virtual Docker (MVD 2013.6.0), Molegro Bioinformatics Solutions, Denmark, 2013; Thomsen and Christensen, 2006). ChemBio3D Ultra 10 [30] was used to draw the 3D structures of different ligands. Ligands were further optimized using a free version of Marvinsketch 4.1.13 (Marvinsketch, version 6.1.0, Chemaxon, Budapest, Hungary; http://www.chemaxon.com, 2013) with MM force field, and saved in Tripos mol2 file format. MolDock score functions were used with a 0.3 A° grid resolution. Prior to the calculation of the MolDock scores of the tested compounds, the MVD software was benchmarked docking ribavirin [11].
